# Nanosecond Laser Fabrication of Hydrophobic Stainless Steel Surfaces: The Impact on Microstructure and Corrosion Resistance

**DOI:** 10.3390/ma11091577

**Published:** 2018-09-01

**Authors:** Mehran Rafieazad, Jaffer Alkarim Jaffer, Cong Cui, Xili Duan, Ali Nasiri

**Affiliations:** Faculty of Engineering and Applied Science, Memorial University of Newfoundland, St. John’s, NL A1B 3X5, Canada; jaj508@mun.ca (J.A.J.); cc3461@mun.ca (C.C.); xduan@mun.ca (X.D.); amnasiri@mun.ca (A.N.)

**Keywords:** hydrophobicity, 17-4 PH stainless steel, nanosecond laser, contact angle, corrosion resistance

## Abstract

Creation of hydrophobic and superhydrophobic surfaces has attracted broad attention as a promising solution for protection of metal surfaces from corrosive environments. This work investigates the capability of nanosecond fiber laser surface texturing followed by a low energy coating in the fabrication of hydrophobic 17-4 PH stainless steel surfaces as an alternative to the ultrashort lasers previously utilized for hydrophobic surfaces production. Laser texturing of the surface followed by applying the hydrophobic coating resulted in steady-state contact angles of up to 145°, while the non-textured coated base metal exhibited the contact angle of 121°. The microstructure and compositional analysis results confirmed that the laser texturing process neither affects the microstructure of the base metal nor causes elemental loss from the melted regions during the ultrafast melting process. However, the electrochemical measurements demonstrated that the water-repelling property of the surface did not contribute to the anticorrosion capability of the substrate. The resultant higher corrosion current density, lower corrosion potential, and higher corrosion rate of the laser textured surfaces were ascribed to the size of fabricated surface micro-grooves, which cannot retain the entrapped air inside the hierarchical structure when fully immersed in a corrosive medium, thus degrading the material’s corrosion performance.

## 1. Introduction

Corrosion is an irreversible oxidation reaction on material due to its interaction with an immediate environment [1]. It is a common problem especially in highly aggressive environments [2], degrading the integrity and lifetime of metal surfaces and causing problems both economically and operationally in engineering applications. External factors such as salinity [3], temperature [4], pH [2] and pollutants [5] influence the rate of corrosion of materials. Fabrication processes such as welding [6] and heat treatment [7] can also affect the corrosion resistance of the metal.

One form of corrosion protection that has been recently gaining significant interest among the research community is the application of hydrophobic properties on materials. Combining corrosion protection with other benefits of hydrophobicity such as anti-icing [8], control of bio-fouling [2], self-cleaning [2], water repellency [9], and reduction of turbulent flow resistance [10], makes this property extremely beneficial for applications in harsh or highly humid environments, such as pipelines, wellhead platforms or power transmission lines.

In nature, there are a variety of hydrophobic and superhydrophobic surfaces such as plants’ leaves [11] and living creatures [12]. A surface with a so-called hydrophobic property has a wet contact angle greater than 90°, while superhydrophobic surfaces have remarkably higher water repellency with a wet contact angle greater than 150° [2,11]. Previous studies have discussed two major controlling factors that dictate the hydrophobicity of the surface, i.e., surface roughness and surface energy [2,13]. It should be noted that surface energy is the main factor to achieve hydrophobicity, which primarily is controlled by the surface’s chemical composition [2]. However, superhydrophobicity cannot be obtained only by minimizing the surface energy. It also requires tailoring the surface roughness [10]. As an example, Nishino et al. [13] reported the lowest surface free energy of any solid to be 6.7 mJ/m^2^ obtained by covering the surface of a glass with regular aligned closest hexagonal packed –CF_3_ groups, but the best-achieved contact angle was reported to be only 119°, which is far from superhydrophobicity [13]. Therefore, hierarchical surface features are crucial requirements to achieve superhydrophobicity [10]. These hierarchical surfaces are composed of micro hills and valleys similar to the surface of a lotus leaf or insect bodies [10,11].

A wide variety of materials has been used so far to create hydrophobic and superhydrophobic properties on their surfaces, including both organic materials, which are commonly hydrophobic by nature and inorganic materials, such as metals with a hydrophilic nature [2]. Also, various processes and techniques have been utilized to fabricate hydrophobic surfaces, such as photolithography [14], laser/plasma treatment [15], chemical etching [16] and layer-by-layer deposition [17]. In general, metals due to their better physical, mechanical, chemical and thermal properties, and the laser treatment technique due to its relatively shorter processing time and being highly reproducible have attracted more interest for the fabrication of hydrophobic and superhydrophobic surfaces [18,19]. 

Superhydrophobic surfaces also provide a corrosion resistance improvement to different types of metallic substrates. Boinovich et al. [20] reported that nanosecond laser treatment of Al surfaces induces hydrophobicity, which in turn enhances resistivity to pitting corrosion in sodium chloride solutions. Yuan et al. [18] similarly analyzed the corrosion characteristics of fluoropolymer films on copper substrates in NaCl medium. It was noted that polymeric films due to the chelating action and formation of insoluble diffusion barriers could protect the substrates from corrosion [19], resulting in 12 times lower corrosion current density for the coated samples than that of the base metal after 21 days of exposure in NaCl corrosive environment [19].

Corrosion resistance evaluation of superhydrophobic stainless steel surfaces has also been investigated in some studies. Latthe et al. [21] studied the corrosion resistance of a chemically etched super hydrophobic 430 stainless steel substrate immersed in 5 wt.% NaCl aqueous solution and described the resultant superhydrophobic surface as a “physical barrier between the metal and the environment”. In a similar experiment, Park et al. [22] used a 304 stainless steel substrate immersed in 3.5 wt.% NaCl aqueous solution to compare superhydrophobic and superhydrophilic variations of the substrate and found that the superhydrophobic surfaces showed a higher resistance to corrosion. A different approach, based on the application of three different superhydrophobic membranes on 080M46 steel, was taken by Wang et al. [23] to fabricate hydrophobic steel surfaces. Similarly, they reported that the corrosion resistivity of all three membranes was far superior to the base metal [23]. Trdan et al. [24] also reported a direct relation between wettability and corrosion behavior by showing the effect of the transition from superhydrophilicity to superhydrophobicity on the corrosion behavior of AISI 316L stainless steel using nanosecond laser treatment. All the above studies indicate that there might be correlations between corrosion resistance and the wettability of metallic surfaces.

As reported by Mohamed et al. [2], in all previous studies the mechanism behind the corrosion resistance improvement of the metallic surfaces due to their hydrophobic and superhydrophobic properties was claimed to be the retaining of the air pocket layer on the surface of the metal, acting as a barrier and preventing the corrosion process from taking place. Air trapping on the surface can enhance the hydrophobicity since air is an extremely hydrophobic compound with a contact angle of 180° [25].

Although a few studies have been carried out on the correlation between hydrophobicity and the corrosion resistance of stainless steel alloys [6,7,26], 17-4 PH stainless steel with broad application in the marine, chemical, petrochemical, food-processing and aerospace industries has never been the focus of such studies. Furthermore, in most of the past studies, highly expensive ultrashort pulse lasers, also known as ultrafast lasers, such as femtosecond and picosecond lasers, have been utilized to fabricate hydrophobic surfaces with prolonged processing time (scanning speed of 1 mm/s) [23]. Very limited information is available on laser fabrication of hydrophobic surfaces and its effectiveness using nanosecond fiber lasers with fast processing time [23]. These lasers would be highly beneficial for surface texturing of materials for real industrial applications and should be considered as a cost-effective alternative to expensive ultrashort lasers. Therefore, the purpose of this study is to evaluate the effectiveness of hydrophobic 17-4 PH stainless steel surfaces fabricated through nanosecond fiber laser surface texturing combined with applying a hydrophobic coating and to analyze its impact on the microstructure and corrosion resistance of the surface.

## 2. Materials and Methods

### 2.1. Material

Martensitic type 17-4 PH stainless steel (SS 630-H1025, McMASTER-CARR, Elmhurst, IL, USA) with a size of 20 × 10 × 3 mm was used as the base metal coupons for laser surface texturing. The H1025 heat treatment designation indicates that the alloy has been precipitation hardened at 551 ± 15 °C for 4 h followed by air cooling. The resultant hardness of the alloy was measured to be ~39 ± 3 HRC (Hardness Rockwell C). The measured chemical composition of the base metal before laser treatment is given in Table 1. 

### 2.2. Preparation of Hydrophobic Surfaces

**Figure 1:**
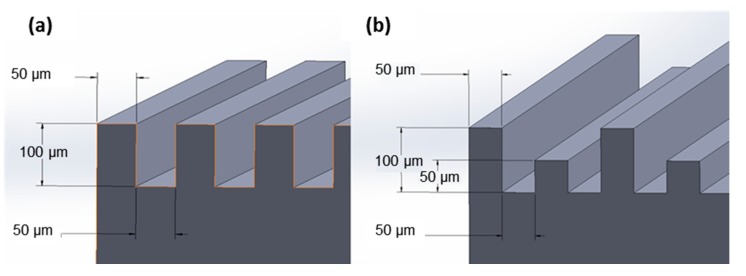
Desired designs of (**a**) the channeled structure and (**b**) the varied channeled structure.

To prepare the hydrophobic surfaces, a combination of laser surface texturing followed by applying a 2–4 nm thick optically clear hydrophobic dip coating, to roughen the surface and lower the surface energy, respectively, was used. The nanoscale thickness of the used hydrophobic coating is reported by the supplier [27]. The surface of the base metal was subjected to mechanical grinding using 600 grit SiC abrasive paper prior to the surface texturing. For the surface texturing, a BMF20A/B fiber laser machine (Shenzhen KEYAN, Shenzhen, China) with the wavelength of 1060 nm, the laser power of 12 W, the frequency of 20 kHz, the scanning speed of 600 mm/s, and the pulse width of 60 ns on a substrate surface of 20 × 10 mm in an argon atmosphere was employed. The chemical composition of the hydrophobic coating was 50–52% Ethanol, mixture of 42–46% 2-(difluoromethoxymethyl)-1,1,1,2,3,3,3-heptafluoropropane and 4-methoxy-1,1,1,2,2,3,3,4,4-nona-fluorobutan, 2–3% 2 propanol, and 2–3% Methanol (Metal Repellency Treatment from Aculon Performance Surface Solutions [27]). To remove oil and other contaminants from the specimens’ surfaces, the stainless steel samples were first cleaned ultrasonically in acetone. After this procedure, three variations of hydrophobic surfaces were produced. The first group of samples was simply coated with the hydrophobic coating (referred to as “coated base”), while the other two variations were textured with the laser first using two different topographical designs as shown in Figure 1 and then were coated using the hydrophobic coating (referred to as “coated channeled” and “coated varied channeled”).

### 2.3. Characterization

The surface structures, morphologies and microstructures of the prepared samples were studied using an optical microscope (Nikon Eclipse 50i, Nikon Instruments Inc., Melville, NY, USA) and a scanning electron microscope (SEM) (FEI MLA 650F, Thermo Fisher Scientific, Hillsboro, OR, USA) equipped with a high-throughput Bruker energy dispersive X-ray (EDX, Bruker, Ringoes, NJ, USA) analytical system, which was used to investigate compositional inhomogeneity and possible alloying elements loss from the regions that encounter superficial melting followed by solidification during the laser surface texturing process. To prepare the samples for microscopic analysis, the samples were mounted in an epoxy resin followed by standard grinding and polishing sample preparation procedures for stainless steels. The polished specimens were then etched using Nital 5% reagent (5 vol.% HNO_3_ and 95 vol.% Methanol) to reveal the microstructure.

### 2.4. Wettability Measurements

Contact angle measurements were performed using an optical-based contact angle measuring system (OCA 15 15EC, DataPhysics Instruments, Filderstadt, Germany) consisting of an adjustable sample support unit, a light source, a dosing syringe, camera and lens under the clean experimental condition to eliminate the contamination of the surfaces via air-born organics. The system’s software captures and analyzes the drop shape and measures the static contact angle. The sample was laid flat on a smooth and clean surface in line with the camera at a distance of approximately 10 cm. The camera’s focus and light source were adjusted for optimal clarity and brightness of images. A water droplet of 10 μL was dosed using the software at a dispense rate of 2 μL/s. The given diameter of the sessile drop is about 1.4 mm. All analyses were carried out at room temperature.

### 2.5. Electrochemical Measurements

Electrochemical corrosion measurements were performed on the treated surfaces using an IVIUM CompactStat™ Potentiostat (CompactStat, Ivium Technologies, Eindhoven, The Netherlands) controlled by the software from the same manufacturer. Using a conventional three-electrode setup, the samples were exposed to aerated 3.5 wt.% NaCl solution to mimic a seawater environment at the temperature of 25 °C. A graphite rod was used as the counter electrode (CE), and saturated silver/silver chloride (Ag/AgCl) was used as the reference electrode (RE). The samples were connected as an electrode using copper wire and conductive tape. The exposed area of the base metal and coated base metal samples was 2 cm^2^, and for the channeled and varied channeled samples the exposed area was 5.96 cm^2^ and 4.97 cm^2^, respectively. The rest of the sample was covered with polyester resin to isolate the surfaces not required for testing. Potentiodynamic polarization analysis was conducted by scanning from −0.3 V to +0.3 V about the open circuit potential (OCP) with a scanning rate of 0.125 mV/s. The repeatability of the corrosion test results was measured by testing at least three samples.

## 3. Results

### 3.1. Surface Morphology

**Figure 2:**
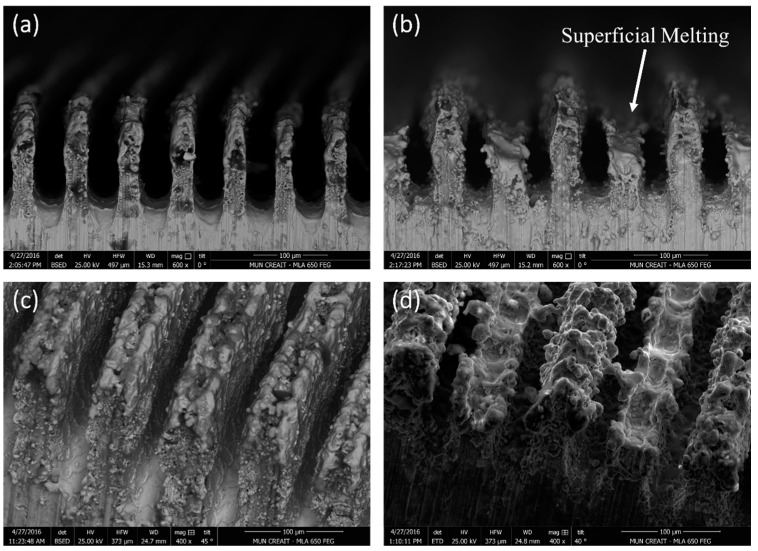
SEM images of (**a**) the channeled structure side view; (**b**) the varied channeled structure side view; (**c**) the channeled structure top view and (**d**) the varied channeled structure top view.

The desired surface topographies for this study have been shown in Figure 1, while the SEM images from the surfaces of the actual fabricated samples from side and top views are presented in Figure 2. The tips are thinner in Figure 2a,c due to the ablation of material by the laser. The shorter tips in Figure 2b,d show the effect of prolonged laser exposure and how the top has flattened due to melting and evaporation of material. There is also evidence of superficial melting followed by solidification (indicated in Figure 2b) of some of the material on the structured surface.

### 3.2. Microstructure

**Figure 3:**
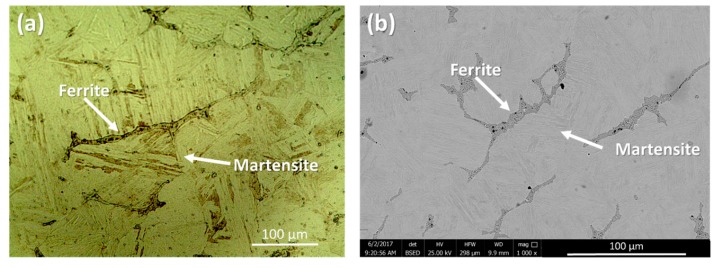
(**a**) Optical microscope image and (**b**) SEM micrograph of the 17-4 PH stainless steel base material.

Figure 3 demonstrates the optical and SEM micrographs of the 17-4 PH stainless steel materials used in this study composed of martensite and δ-ferrite phases. The parallel groups of lath martensite as the matrix and elongated δ-ferrite phase in different directions along the primary austenite grain boundaries form the microstructure of the base metal.

As shown in Figure 3, the ferrite phase encompasses very fine precipitates, which were detected exclusively on the ferrite phase. Although no evidence of secondary phase formation from the martensitic phase was detected under SEM, the applied H1025 heat treatment should have triggered the formation of coherent nano-precipitates, i.e., Cu-rich precipitates (CRPs), from the martensite phase [28,29].

**Figure 4:**
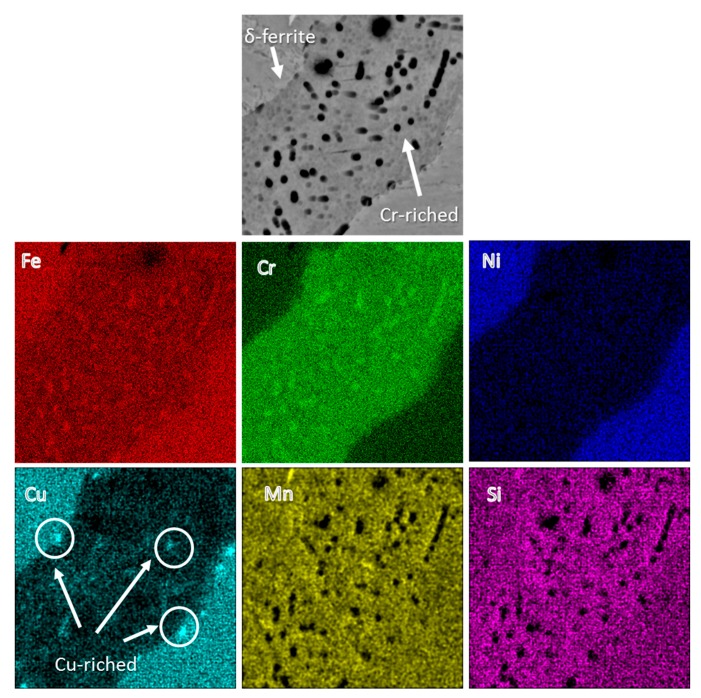
EDX concentration maps of the δ-ferrite phase.

The EDX concentration maps of the ferrite phases in Figure 4 indicate a higher concentration of Cr in spherical precipitates within the δ-ferrite region [30]. Park et al. [31] reported that Cr-enriched phases deteriorate the corrosion resistivity of the alloy. As shown by the white arrows in the Cu map concentration, a few irregularly shaped Cu-enriched particles (CRPs) within the ferrite phase are also present. In comparison, the martensite phase showed a uniform concentration of Cr and Cu elements. Table 2 shows EDX compositional analysis results of the major micro-constituents of the sample, including both ferrite and martensite phases.

### 3.3. Wetting Behavior

**Figure 5:**
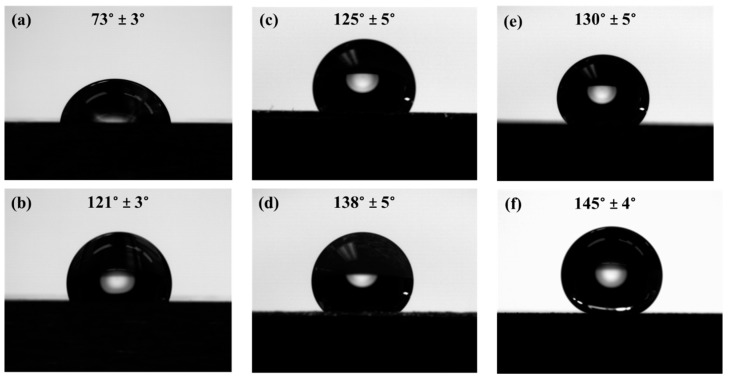
Contact angle measurements of the (**a**) base without coating; (**b**) coated base; (**c**) channeled without coating; (**d**) coated channeled; (**e**) varied channeled without coating; and (**f**) coated varied channeled surfaces.

The static contact angles for the fabricated surfaces are shown in Figure 5. As expected, the base metal surface has the lowest contact angle (see Figure 5a). The coated base sample shows an increase of 48° contact angle compared to the base metal indicating a hydrophobic surface. The channeled and varied channeled structures (Figure 5c,e) show higher hydrophobicity (~4° and 9° increase, respectively, from the coated base sample) primarily due to the surface micro-roughness that was fabricated on the surface using the laser-texturing process. After applying the hydrophobic coating on the textured surfaces, the channeled and varied channeled surfaces exhibited more hydrophobic behavior with contact angles of 138° ± 5° and 145° ± 4°, respectively. Therefore, the combined effects of surface micro-roughness and the coating have resulted in a hydrophobic surface with contact angles just below the required contact angle for superhydrophobicity (150°).

**Figure 6:**
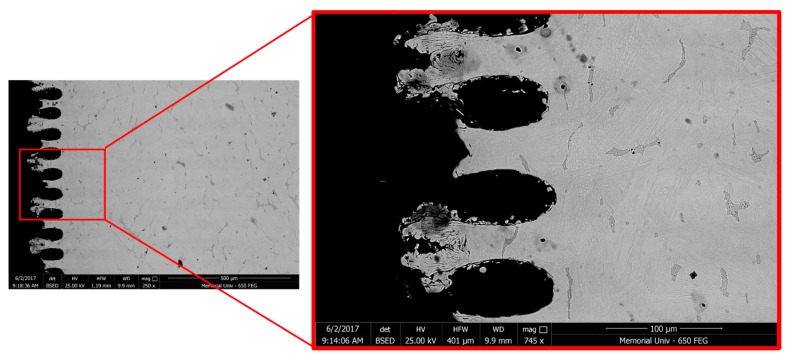
Surface features of the sample with varied channeled morphology.

As evidenced by Figure 5, the varied channeled sample with or without a coating shows a higher contact angle than its channeled counterpart. To fabricate the varied channeled samples, the laser beam was directly interacting with the surface of the channels to adjust the height of the channels relative to each other. This has resulted in the superficial melting of the tips followed by their rapid cooling and solidification, leading to the formation of solidified regions on the tips of the channels (shown in Figure 2d and Figure 6). These regions provide multimodal roughness on the fabricated surface, contributing to further improvement of the hydrophobicity. Similar phenomena were reported in previous studies [23]. When additional smaller sized surface features are added to the existing surface structure, the water repellency and the contact angle can be increased [23]. However, in the channeled sample, the channels’ tips do not experience melting and solidification, and less additional surface features were generated, resulting in a lower contact angle than the varied channeled sample.

In the context of surface wettability, there are two models capable of describing the correlation between surface roughness and hydrophobicity. The first one proposed by Wenzel [32] assumes that the liquid droplet is in contact with the absolute area of the rough surface, by

(1) $$\;cos\theta =rcos\theta_{0}\;$$

where *θ* shows the contact angle on the rough surface, *θ*_0_ represents the equilibrium contact angle on an ideal smooth surface, and *r* defines the non-dimensional surface roughness ratio, also known as the topography factor [32] When this model is applied to rough surfaces, it predicts an increase of the apparent contact angle (*θ*) for hydrophobic surfaces and a decrease of the contact angle for hydrophilic surfaces by increasing the surface roughness (*r*-value). Because the laser surface texturing in this study changed the wettability of the surface from a hydrophilic surface (*θ* = 73° ± 3°) to a hydrophobic surface (*θ* > 90°), the Wenzel model is not expected to be able to describe the behavior of the fabricated laser textured surfaces.

The other model is the Cassie-Baxter model [33], in which it is assumed that droplets do not wet the rough surfaces completely due to the existing air packs that are trapped within the interstices of the rough surface. The apparent contact angle ($\theta_{r}^{C}$) with this model is calculated by [26]:

(2) $$\;cos\theta_{r}^{C}=f(cos\theta_{e}+1)-1\;$$

where *f* is the fraction of the surface in contact with the liquid and $\theta_{e}$ is the intrinsic contact angle for a smooth surface. Applying this model to both hydrophobic and hydrophilic surfaces confirms that the apparent contact angle on rough surfaces is always higher than that of smooth surfaces [34]. Therefore, the measured contact angles in this study are expected to fit the Cassie-Baxter model and not the Wenzel model.

### 3.4. Corrosion Behavior

**Figure 7:**
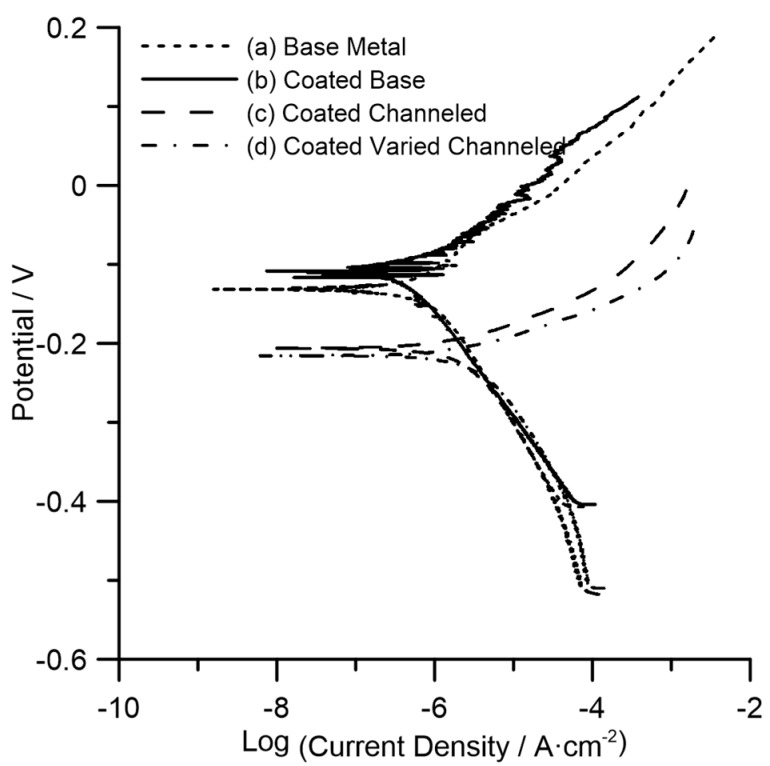
Tafel plot comparison among the (**a**) base metal; (**b**) coated base; (**c**) coated channeled and (**d**) coated varied channeled surfaces.

Figure 7 shows the polarization characteristics (Tafel plots) of all samples comparing the base metal with the hydrophobic surface variants. As a general trend, better corrosion resistance is shown by an increase in corrosion potential and a decrease in corrosion current density. Hence, the preliminary observation of the graph shows that the base metal and coated base metal show higher corrosion resistance than the more hydrophobic textured samples.

From the graph, each plot can be considered to calculate and quantify the corrosion rate. The anodic slope (*β_A_*) and the cathodic slope (*β_C_*) can thus be obtained for each plot. The intersection between the two slopes indicates the corrosion current (*I_CORR_*) and the corrosion potential (*E_CORR_*) versus the reference electrode. However, this can also be calculated (in SI units) from the following formula:

(3) $$\;I_{CORR\;}=\left(\beta_{A}\;\beta_{C}\right)/\left(2.3\times R_{p}\times \left(\beta_{A}+\beta_{C}\right)\right)$$

where *R_p_* is the polarization resistance (in ohms·cm^−2^) measured by the potentiostat software. The corrosion current can then be used to calculate the corrosion rate (in mm/year) from the following formula:

(4) $$\;\mathrm{Corrosion}\;\mathrm{Rate}=\left(3.27\times {10\;}^{3}\times I_{CORR}\times E.W.\right)/\left(A\times \rho \right)$$

where *E.W.* stands for the equivalent weight of the alloy (in grams), *A* is the surface area (in cm^2^) exposed to the electrolyte, and ρ is the density of the sample (in g/cm^3^).

Table 3 shows the corrosion potential, corrosion current, corrosion current density, and the corrosion rate of each of the samples calculated from the formulas above. The corrosion rate of the base metal shows an evident decrease from 0.012 mm/year to 0.005 mm/year with applying the hydrophobic coating, representing an almost 50% increase in its uniform corrosion resistance, which is ascribed to the protective nature of the coating on the substrate. On the other hand, the corrosion rate of the coated channeled and coated varied channeled surfaces was increased to 0.032 mm/year and 0.043 mm/year, respectively, representing a loss of corrosion resistance by a factor of ~3–4, even though the coated textured surfaces were more hydrophobic as evidenced by showing higher contact angles (Figure 5). As shown in Table 3, the corrosion current density values (*I_CORR_*) of the hydrophobic laser-textured surfaces follow the same trend as the corrosion rate data since *I_CORR_* is directly related to the corrosion rate (see Equation (4)). However, the corrosion potential values (*E_CORR_*), correlating to the thermodynamic tendency for corrosion show a pronounced negative shift to less noble values for the laser-textured surfaces as compared to those of the base metal representing a higher corrosion tendency. Therefore, coated laser-textured surfaces displayed both an increased corrosion rate and corrosion tendency, meaning water repellency unexpectedly was not found to be effective towards protection of the surface against a uniform corrosion attack in this study.

**Figure 8:**
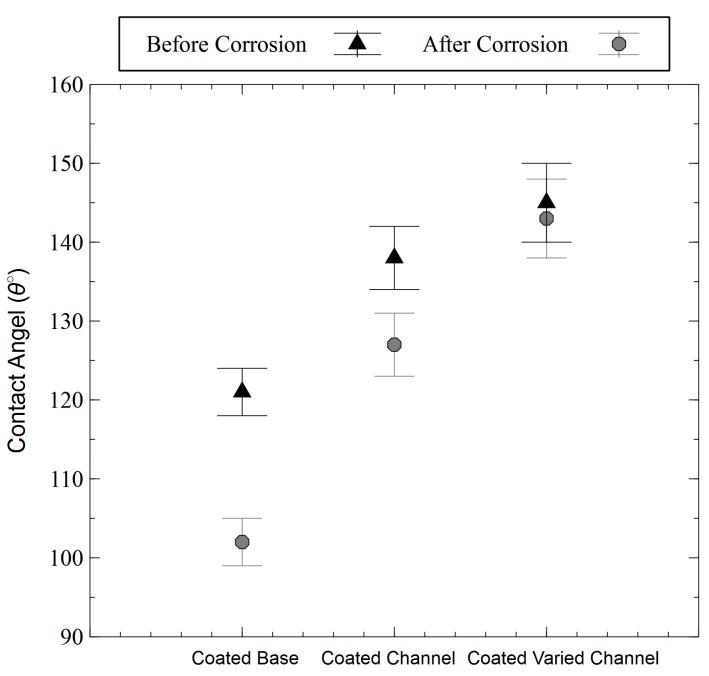
Contact angle (in degrees) comparison before and after the potentiodynamic polarization tests.

Contact angle measurements after the corrosion tests were also performed to check if the coating was compromised during the corrosion test. Figure 8 shows that although surfaces are still hydrophobic, there is a slight decrease in the contact angle values, which is more pronounced for the coated base metal. After fully immersing the samples in the corrosive electrolyte and performing the potentiodynamic polarization testing, the hydrophobic coating might have been locally peeled off from the surface resulting in a decrease in the contact angle.

**Figure 9:**
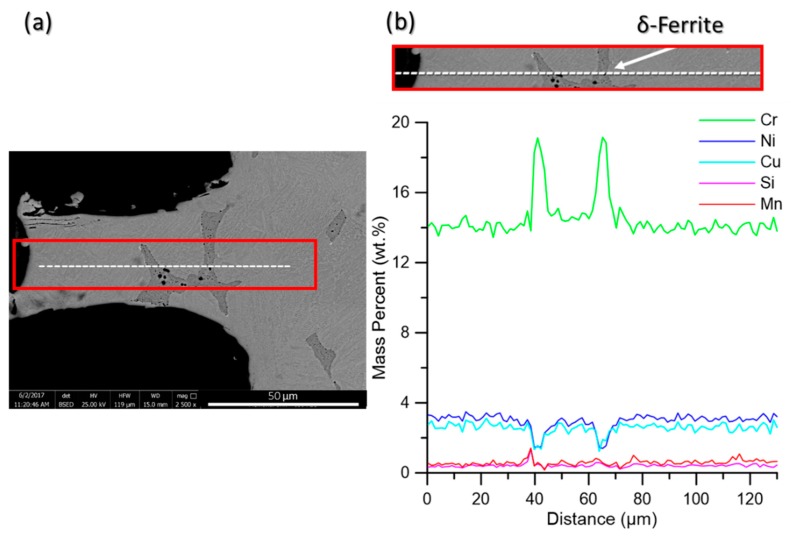
(**a**) SEM image taken from one surface feature of the sample with varied channeled morphology showing the position of the EDX-line scan using a dashed-line in the enclosed area, and (**b**) the corresponding EDX composition line scans indicating scans of Cr, Ni, Cu, Si, and Mn.

As mentioned above, hydrophobicity and the resulting corrosion resistivity enhancement are primarily controlled by two main factors, namely surface morphology and surface energy, and the inferior is controlled by surface chemical composition [2]. During the laser surface texturing, the interaction of the laser with the surface and the resultant high temperature on the surface melts and ablates materials, which can also cause an undesirable loss of volatile alloying elements from the superficial melted regions. Consequently the metallurgical properties of the surface including its electrochemical resistance can change. To investigate the impact of the laser surface texturing on chemical composition inhomogeneity and possible loss of volatile elements from the melted regions (Figure 9a), SEM-EDX composition line scan analysis of the melted regions was performed. As Figure 9b shows, there is no elemental deficiency in the melted region adjacent to the surface relative to the base metal, especially Chromium and Nickel elements, which are primarily responsible for the corrosion resistivity of the alloy [35]. This confirms that the performed laser surface texturing had no detrimental effect on the chemical composition of the material after melting followed by solidification, which will be in contact with the corrosive medium. Therefore, the reduction of corrosion resistivity of the laser textured specimens is not due to the elemental loss from the superficial melted regions or any change in the substrate’s chemical composition.

As shown in Figure 9b, two peaks were detected across the Cr line scan, in the same positions as another two valleys across the Ni and Cu lines, in which their positions correspond to the δ-ferrite phase containing higher Cr content and a lower concentration of Ni and Cu relative to the martensitic matrix. Delta ferrite phase has been shown to have a higher solubility for Cr, since Cr is a ferrite stabilizing element and lower solid solubility for the Ni and Cu elements [36].

The other controlling factor to be investigated is the surface morphology. The mechanism behind the improved corrosion resistivity of superhydrophobic stainless steels surfaces in previous studies [37] has been reported to be the trapped air (air-packs) within the micro/nano-structures that hinders the solution penetration into the surface (as proposed by the Cassie-Baxter’s model [33]), resulting in a reduced contact area between the surface and the corrosive medium, leading to the improved corrosion resistivity of the surface. Therefore, for the case of superhydrophobic surfaces, the surface features are small enough to retain the trapped air on the surface even after full immersion of the sample in the corrosive medium during electrochemical testing. This requires careful optimization of the size of grooves and surface protuberances. Luo et al. [38] reported that although having microfeatures can generate hydrophobic properties, superhydrophobicity can be achieved only if the feature’s ratio is optimum. They noted that if the width of features decreases from 250 µm to 10 µm, the contact angle will be increased from 60° to 129° [38].

The presented results in this study demonstrated that when the laser-textured stainless steel surface is only hydrophobic, the surface features are not small enough to maintain the protective air-packs between the surface and the solution. Therefore, all the trapped air might have escaped from the surface as soon as the surface is immersed in the corrosive medium. Consequently, all the surface micro-grooves will act as a channel for a microfluidic, resulting in an increased area of solid liquid interface and an enhanced corrosion rate. Similar corrosion resistance degradation was reported by Trdan et al. [24] for the laser-surface-textured 316L stainless steel when the surface is not superhydrophobic. They observed a significant directional corrosion attack propagated inside the channels [24]. Contrarily, this effect is entirely annihilated when the surface becomes superhydrophobic [24].

Although the applied hydrophobic coating contributed to the water-repelling property of the surface and a slight improvement in the corrosion performance of the base metal, the size of the nanosecond-laser-fabricated micro-grooves was found to be the primary factor in dictating the corrosion performance of the hydrophobic surfaces. Therefore, nanosecond fiber laser surface texturing will be an effective replacement for slow and highly expensive ultrashort laser texturing, only if the size of micro-groove features is carefully optimized, resulting in the superhydrophobic property, which allows the air-packs to remain on the surface inside the hierarchical structure in a fully immersed condition. Otherwise, the resultant corrosion loss compromises the effectiveness of this technique.

## 4. Conclusions

Hydrophobic 17-4 PH stainless steel surfaces were successfully fabricated using a fast and cost-effective nanosecond laser surface texturing technique followed by applying a hydrophobic coating to the surfaces. This processing technique facilitates the fabrication of large-scale hydrophobic metallic surfaces proper for various industrial applications. The samples showed hydrophobic properties directly after the fabrication process. The obtained hydrophobic property can be ascribed to the combined effects of the laser-induced roughness and low surface energy resulting from the coating. The coating could only increase the steady state contact angle of water on the non-textured base metal up to 121°; however, by laser texturing of the surface, the contact angle was increased up to 145° without undesirable loss of volatile elements from the melted zones.

The resultant hydrophobicity did not render the desired corrosion protection capability to the stainless steel substrate. The lowest corrosion potential with the highest corrosion current density leading to the highest corrosion rate was measured on the laser-textured hydrophobic samples. Therefore, the water-repelling property of the 17-4 PH stainless steel was not found to be effective in preventing the aggressive chloride ions from approaching the substrate. This can be attributed to the large size of the fabricated micro-grooves on the surface, meaning the surface features are not capable of retaining the entrapped air inside the hierarchical structure in a fully immersed condition in the corrosive medium, resulting in an increased contact area between water and the solid substrate and degradation of the corrosion property. Nonetheless, the hydrophobicity of the fabricated samples was maintained even after electrochemical corrosion testing. Therefore, further optimization of the size of the micro-grooves is required to create a superhydrophobic surface with an enhanced corrosion property.

## Figures and Tables

**Table 1:** The measured chemical composition of 17-4 PH stainless steel before laser treatment (all in wt.%)

Elements	Cr	Ni	Cu	Si	Mn	Fe
17-4 PH	16.70 ± 0.05	3.70 ± 0.06	2.91 ± 0.07	0.26 ± 0.08	0.46 ± 0.01	Bal.

**Table 2:** The measured chemical composition of 17-4 PH stainless steel micro-constituents (all in wt. %).

Phase	Cr	Ni	Cu	Si	Mn	Fe
δ-Ferrite	22.3 ± 0.2	1.6 ± 0.1	1.2 ± 0.2	0.4 ± 0.1	0.1 ± 0.1	Bal.
Martesite	16.5 ± 0.1	3.7 ± 0.1	2.9 ± 0.1	0.3 ± 0.1	0.1 ± 0.9	Bal.

**Table 3:** Results obtained and calculated from the Tafel plots shown in Figure 7.

Surface Type	Corrosion Potential (V_Ag/AgCl_)	Corrosion Current (A)	Corrosion Current Density (A·cm^−2^)	Corrosion Rate (mm/y)
Base metal	−0.136	2.070 × 10^−6^	1.035 × 10^−6^	0.012
Coated base metal	−0.110	8.036 × 10^−7^	4.018 × 10^−7^	0.005
Coated channeled	−0.209	1.657 × 10^−5^	2.776 × 10^−6^	0.032
Coated varied channeled	−0.205	1.878 × 10^−5^	3.799 × 10^−6^	0.043
